# Biomarkers and therapeutic targets in early Alzheimer’s disease: an Olink proteomics study

**DOI:** 10.3389/fneur.2025.1615152

**Published:** 2025-07-17

**Authors:** Jianan Wei, Hui Ni, Weiwei Liu, Hui Xu, Chen Wang, Geng Geng, Zhongwei He, Guofang Chen

**Affiliations:** ^1^The Affiliated Xuzhou Clinical College of Xuzhou Medical University, Xuzhou, Jiangsu, China; ^2^Department of Neurology, Xuzhou Central Hospital, Xuzhou, Jiangsu, China

**Keywords:** Alzheimer’s disease, dementia, biomarkers, Olink proteomics, inflammation

## Abstract

**Purpose:**

This study aims to analyze differential expression of inflammation-related proteins in plasma from patients with mild cognitive impairment (MCI) and early-stage Alzheimer’s disease (AD), for exploring potential biomarkers and therapeutic targets for AD, providing new possibilities for the early diagnosis of AD and the theoretical basis for the subsequent targeted therapy.

**Participants and methods:**

The study included 30 adults: 10 healthy control subjects (HC group), 10 patients with AD [AD group, positron emission tomography (PET)-confirmed] and 10 with MCI (MCI group, PET negative). We carried out Proximity Extension Assay (PEA) on 92 inflammation-related proteins in the plasma samples of these 30 participants by using the Olink proteomics technology. Subsequently, to evaluate the clinical translational potential of these Differentially Expressed Proteins (DEPs) as early AD biomarkers and their potential mechanisms of action, we performed Receiver Operator Characteristic (ROC) curve analysis and functional enrichment analysis on these proteins.

**Results:**

Compared with the HC group, uPA, CX3CL1, CDCP1, Flt3L, SCF and TWEAK proteins were significantly upregulated in AD group, with Area Under the ROC Curve (AUC) values of 0.96 (*p* = 0.001), 0.90 (*p* = 0.002), 0.87 (*p* = 0.005), 0.77 (*p* = 0.018), 0.89 (*p* = 0.003) and 0.75 (*p* = 0.041), respectively, and cutoff values of 10.083 pg./mL, 3.411 pg./mL, 3.391 pg./mL, 9.038 pg./mL, 8.984 pg./mL and 8.998 pg./mL. Similarly, in MCI group, uPA and CDCP1 also exhibited upregulation, with AUC values of 0.92 (*p* = 0.001) and 0.83 (*p* = 0.013), and cutoff values of 10.133 pg./mL and 3.803 pg./mL, respectively. These DEPs may be implicated in pathological processes such as neuroinflammation, neuronal death, and synaptic dysfunction.

**Conclusion:**

Using Olink proteomics technology, this study identified several plasma inflammatory proteins associated with AD, which were proposed as potential biomarkers for early diagnosis. While these findings provided novel insights into early AD screening and molecular mechanisms, and suggested possible therapeutic targets, several limitations were noted. The study’s modest sample size and cross-sectional design limited the ability to assess dynamic changes in these biomarkers during disease progression. Future large-scale longitudinal studies should validate their clinical utility.

## 1 Introduction

AD, the most prevalent form of senile dementia, exhibits complex etiology and insidious neuropathological changes beginning 10–20 years before clinical onset (1). MCI, a transitional stage between normal aging and dementia, represents a critical window for early intervention (2). With 16.99 million AD cases in China (2021) and global prevalence projected to rise from 55 million (2019) to 139 million (2050) (3, 4), AD poses a growing societal and economic burden demanding urgent solutions.

In 2024, the National Institute on Aging-Alzheimer’s Association (NIA-AA) updated the Amyloid/Tau/Neurodegeneration (ATN) diagnostic framework for AD based on the 2018 criteria (5). In addition to core Aβ, tau fluid, and PET biomarkers non-specific inflammatory biomarkers [such as Glial Fibrillary Acidic Protein (GFAP), etc.] were also included, and it was proposed that they could also be used for staging, prognosis or as indicators of biological therapeutic effects. At the same time, it was proposed that non-specific neuroinflammatory biomarkers (such as microglia) were significant for the study of AD. This indicates that inflammatory response has the potential to reflect the progression of AD.

In recent years, blood-based biomarkers for AD have garnered significant attention due to their accessibility, convenience, minimal invasiveness (6). Among them, indicators such as plasma Aβ42, p-tau217, p-tau181, p-tau231, etc. have been included as core biomarkers (5) category for AD diagnosis. While they are regarded as major breakthroughs in AD research, their clinical applications still face several limitations. For instance, low concentrations in plasma result in insufficient sensitivity for reliable detection (6, 7), while differential responses in AD subgroups (particularly APOE ε4 status) restrict clinical generalizability (8). Furthermore, due to variability in detection methods and lack of standardized assays, the specificity of Aβ and tau protein levels still requires improvement, which restricts their application in early AD screening. Therefore, there is an urgent need to explore alternative detection methods and novel biomarkers for AD.

For a long time, it has been widely believed that neuroinflammation is the consequence of AD. However, relevant studies have shown that neuroinflammation not only triggers the disease in the early stage of AD, but also exacerbates the disease progression and runs through the entire course of the disease (9, 10). Neuroinflammation is now recognized as a pivotal driver of Alzheimer’s disease pathogenesis, operating through a self-reinforcing cycle with amyloid-beta (Aβ) and tau pathologies (11). Activated microglia and astrocytes release pro-inflammatory cytokines (e.g., IL-1β, TNF-*α*), which not only accelerate neuronal damage but also directly promote Aβ aggregation and tau hyperphosphorylation (12). PEA technology overcomes historical limitations in inflammatory biomarker research, offering exceptional sensitivity down to the fg/mL level and a dynamic range spanning 10 orders of magnitude, covering high-, medium-, and low-abundance proteins (13). By leveraging Olink technology, we can perform qualitative and quantitative analyses of dynamically changing protein profiles in biological systems, making it highly valuable for disease proteomics research. At present, in the application of proteomics in AD research, it is mainly based on brain tissue or cerebrospinal fluid (CSF) (14). Although CSF can provide direct evidence for the central pathological changes of AD (15, 16), its invasiveness makes large-scale population screening and dynamic monitoring difficult to implement (17). By contrast, plasma inflammatory markers showed superior analytical performance (wider dynamic range, improved stability) and more complete pathological representation of AD neuroinflammation compared to Aβ/tau biomarkers (18). Therefore, exploring proteomic plasma markers related to inflammation might be an important approach for the diagnosis, evaluation and disease monitoring of AD. Besides, we hypothesize that plasma inflammatory proteins measured by Olink PEA technology can discriminate early-stage AD/MCI from cognitively unimpaired controls, reflecting neuroinflammatory processes in preclinical AD.

Based on current knowledge, few studies have systematically investigated differential expression of inflammation-related proteins between healthy individuals and early-stage AD patients (19, 20). This study aims to utilize Olink proteomics technology to profile plasma inflammation-related protein alterations in early-stage AD patients, evaluate their potential as diagnostic biomarkers through comprehensive statistical validation, and preliminarily investigate their mechanistic implications and therapeutic targeting potential in AD pathogenesis.

## 2 Materials and methods

### 2.1 Participants inclusion, informed consent

This study was conducted between July 2023 and July 2024, which was approved by the Ethics Committee of Xuzhou Central Hospital (Approval No.: XZXY-LK-20230718-0109), and all participants or their family members agreed to and signed the informed consent form. A total of 30 samples were included in this study, including 20 patients with cognitive decline, all of these patients completed Florbetapir F 18-PET examination at the PET center of Huashan Hospital affiliated with Fudan University, and the results showed that 10 Aβ-positive (AD group) and 10 Aβ-negative cases (MCI group, clinically diagnosed). The remaining 10 participants comprised the healthy control group (HC), recruited from the hospital’s health examination cohort and individually matched to cases by age, sex and BMI. Blood samples were collected from all 30 participants, and plasma was subjected to Olink proteomic analysis to evaluate inflammatory factors.

The healthy control enrollment criteria employed rigorous medical history collection and Mini-Mental State Examination (MMSE). Evidence-based medical research has demonstrated that MMSE exhibits consistent diagnostic efficacy in AD screening (21). Although this cohort did not undergo molecular imaging verification such as Aβ-PET or tau-PET, previous studies have shown a low PET positivity rate in such populations (22). Therefore, the current study considers that these individuals are unlikely to present AD-related pathological changes on Aβ-PET imaging at this stage.

Inclusion criteria for patients with cognitive decline: (1) Unrelated to acute events, as evidenced by the patient’s subjective expression or confirmation by informants, the patient’s cognitive function shows a slow decline and has lasted for ≥6 months; (2) Non-illiterate individuals with the ability to undergo neuropsychological testing; (3) MMSE scores between 21 and 28 points; (4) Age≥55 years when signing written informed consent.

Exclusion criteria for cognitive impairment patients: (1) History of stroke; (2) Major neurological disorders affecting the central nervous system (CNS) other than AD, including but not limited to other forms of dementia, severe intracranial infections (e.g., encephalitis), Parkinson’s disease, multiple concussions, or seizure disorders; (3) Magnetic resonance imaging (MRI) shows major hemorrhage or severe cerebral leukoencephalopathy; (4) Diagnosis of schizophrenia or other severe psychiatric disorders; (5) Severe/unstable diseases, including cardiovascular/cerebrovascular diseases, hematologic disorders, respiratory insufficiency, hepatic/renal dysfunctions, with expected expectancy <24 months; (6) Illiterate (e. g., unable to read/write, recognize symbols); (7) PET contraindications.

### 2.2 Plasma sample collection

Fifty milliliters of peripheral venous blood were collected from each of the 30 participants using purple EDTA anticoagulant tubes. Within 1 h after collection, the samples were centrifuged at a speed of 3,000 r/min for 10 min at 4°C. Subsequently, the centrifuged samples were divided into 3–4 Eppendorf tubes, which were then stored at −80°C refrigerator for subsequent testing, with 1 freeze–thaw cycle.

### 2.3 Inflammation-related protein analysis

The Olink Target protein assay is based on PEA and is carried out with the help of the Fluidigm Q-PCR platform for high-throughput analysis. The Olink Target® 92 Inflammation Panel (Olink Proteomics, Whale Voyage Gene Technology Co. Ltd., Shanghai, China) strictly follows the manufacturer’s guidelines achieve precise quantification of protein levels. This panel targets 92 inflammatory-related proteins, including: Cytokines (e.g., IL-6, TNF-*α*, IFN-*γ*), Chemokines (e.g., CCL2/MCP-1, CXCL10/IP-10), Growth factors (e.g., VEGF-A, G-CSF), etc. Utilizing PEA technology, this platform employs matched antibody pairs to specifically bind target proteins and generate amplifiable DNA barcode signals proportional to protein concentration. The results are reported as Normalized Protein Expression (NPX) values. Its values are scaled in the Log2 scale, where high NPX values correspond to high protein concentrations. During data analysis, for involving three groups, we performed ANOVA (Analysis of Variance): in a single analysis scenario, after ANOVA model fitting was completed, a global F-test was performed using the ANOVA () function in R software; when the *p*-value was less than 0.05, a post-hoc test analysis was further implemented using Tukey’s method, and the mean value was estimated using the emmeans package in R. Besides, we performed t-test analyses for pairwise comparisons, for proteins showing significant differences, we obtained adjusted *p*-values using the Benjamini-Hochberg method. Regarding the analysis of DEPs, we carried out the related work with the help of Olink Analyze R package (version 3.1.0) and performed Gene Ontology (GO) and Kyoto Encyclopedia of Genes and Genomes (KEGG) enrichment analysis. Meanwhile, heatmaps and volcano plots were produced using the ggplot2 software package, and the correlation between the two protein expressions was explored by Spearman correlation analysis. In addition, the protein–protein interaction (PPI) network of DEP was constructed and presented visually by the online STRING (version11.5).

### 2.4 Data analysis

SPSS (version 27.0) and R software were used for data analysis in this study, with a *p*-value of 0.05. For quantitative data, normality and homogeneity of variance were tested first. Data conforming to both normal distribution and homogeneity of variance were presented as mean ± standard deviation (x ± s), and one-way anova was employed for intergroup comparisons. When overall differences reached statistical significance, pairwise comparisons were performed using Turkey method. If they did not conform to normal distribution, median (M) and interquartile range (P25, P75) are used, the Kruskal-Wallis H test was used, followed by Dunn’s test for significant results. Qualitative data were expressed as frequencies and percentages (*n*, %), with chi-square tests used for intergroup comparisons. Fisher’s exact test was applied when expected frequencies were <5. ROC curve analysis was used to determine cutoff values for continuous variables. Diagnostic ability was assessed using the AUC, with a range from 0 to 1. In the ROC curve analysis, we determined the cutoff values for continuous variables based on the maximum Youden index.

## 3 Results

### 3.1 General data characteristics of the three groups of patients

**Figure 1:**
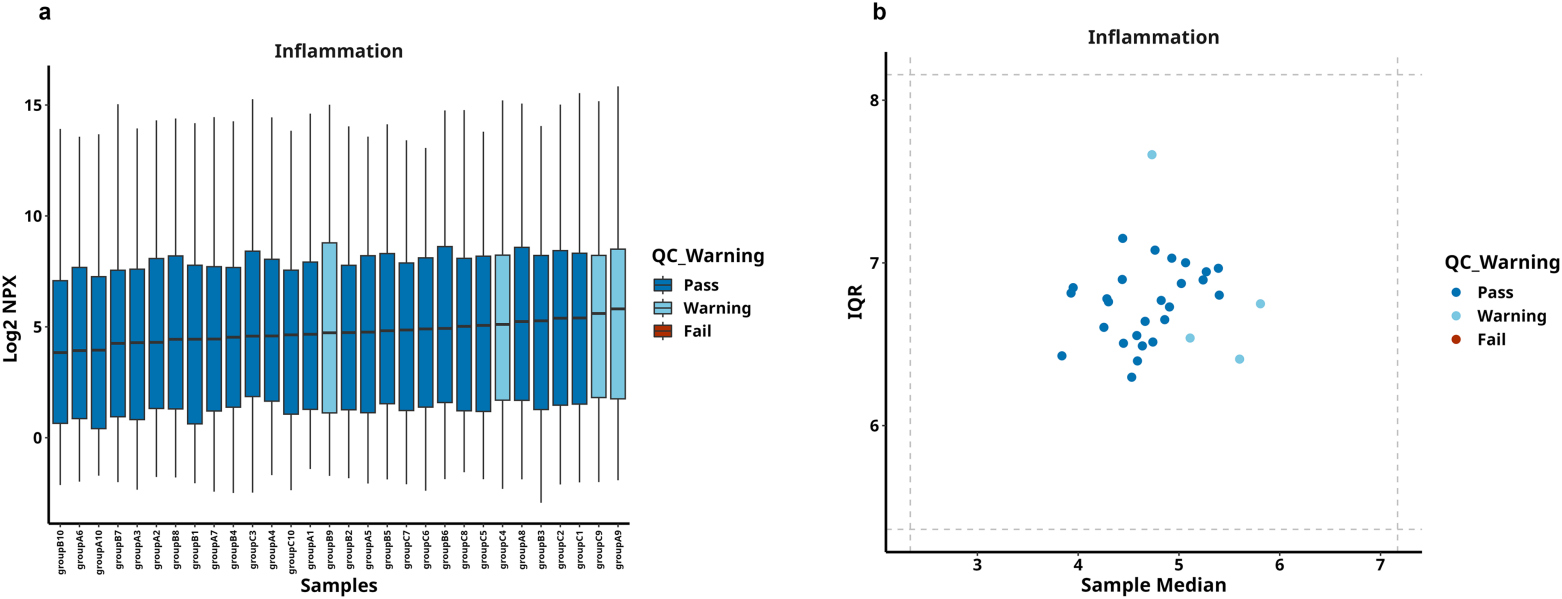
Sample QC distribution results. **(a)** Illustrates the distribution of NPX values for 30 samples. **(b)** Demonstrates the NPX distribution of protein expression levels in these 30 samples, red indicates failed samples, blue represents passed samples, and light blue denotes warning samples.

The general clinical baseline data of all participants were analyzed, and the results were shown in Table 1. The analysis showed that there was no statistical significance in terms of gender, age, height, and body mass index, etc. In addition, an in-depth analysis of the blood counts was carried out, among all metrics, only Monocytes showed statistical significance, while others did not show any statistical differences. The quality control (QC) results, defined as the percentage of samples with measurable protein levels, indicated an 83% detection rate, Figure 1 demonstrated the distribution of NPX in the samples.

**Table 1:** Baseline characteristics of the study population.

Characteristic	Groups			F/χ^2^	*p-*value
	AD (*n* = 10)	MCI (*n* = 10)	HC (*n* = 10)		
Sex				0.27	0.870
Male	(4, 40%)	(5, 50%)	(4, 40%)		
Female	(6, 60%)	(5, 50%)	(6, 60%)		
Age	72.20 ± 4.00	69.10 ± 7.28	66.40 ± 3.41	3.14	0.061
High	1.64 ± 0.58	1.62 ± 0.05	1.65 ± 0.06	0.54	0.592
Weight	63.90 ± 6.59	67.20 ± 12.00	64.80 ± 5.26	0.28	0.760
BMI	23.41 ± 1.75	24.56 ± 1.94	23.68 ± 0.45	1.09	0.362
RBC	4.25 ± 0.32	4.32 ± 0.41	4.42 ± 0.31	0.57	0.551
Platelets	175.00 ± 56.51	185.60 ± 57.47	222.30 ± 40.73	2.27	0.121
WBC	5.49 ± 1.43	5.12 ± 1.29	5.62 ± 1.04	0.37	0.690
Neutrophil	3.25 ± 0.75	3.29 ± 0.99	2.95 ± 1.06	0.40	0.672
Lymphocyte	1.84 ± 0.88	1.45 ± 0.51	2.03 ± 0.43	2.11	0.143
Monocytes	0.33 ± 0.15^*^	0.28 ± 0.12^#^	0.46 ± 0.16	4.20	0.033
Education	10.50 (9.00,13.00)	9.00 (7.00,10.00)	12.00 (9.00,12.00)	2.85	0.241
MMSE	24.40 ± 1.71^*^	25.40 ± 1.90^#^	28.30 ± 1.06	10.99	0.010

AD, Alzheimer’s disease, MCI, Mild cognitive impairment, HC, Healthy Control, BMI, Body Mass Index; RBC, Red Blood Cell; WBC, White Blood Cell, MMSE, Mini-Mental State Examination, with statistical significance observed at *p* < 0.05. MMSE, Mini-Mental State Examination. Intergroup demographic and clinical differences were compared using one-way ANOVA, post hoc Tukey’s test, Kruskal-Wallis H test or chi-square tests as appropriate. ^*^p < 0.05 AD vs. HC; ^#^*p* < 0.05 MCI vs. HC (one-way ANOVA with Tukey’s post hoc test).

### 3.2 Overall differences among the three groups

**Figure 2:**
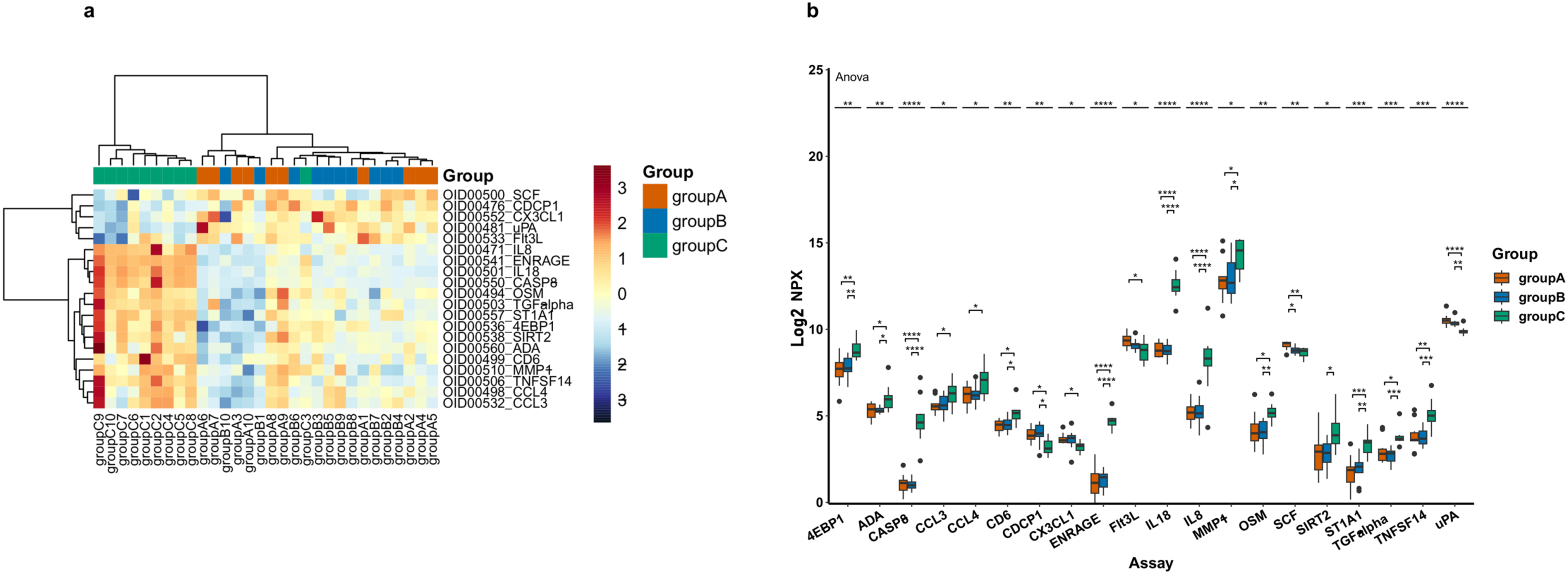
DEPs levels in three patient groups, where A represents AD, B represents MCI and C represents HC. **(a)** Heatmap of DEPs, orange represents group A, blue means group B and green means group C, darker colors indicate higher values, while lighter colors denote lower values. **(b)** Box plot representation of 20 significantly different proteins across groups (the asterisk symbols (*) represents statistically significant differences as determined by one-way-Anova, * < 0.05, ** < 0.01, *** < 0.001, **** < 0.0001).

We measured a total of 92 plasma inflammatory proteins, among which 20 proteins showed significant differences among the three groups (one-way ANOVA, *p* < 0.05; see Table 2 for details). The heatmap showed the hierarchical clustering of the DEPs (Figure 2a) and presented the expression patterns of all significantly different proteins among the three groups using box plots (Figure 2b). Subsequently, we performed pairwise comparisons (AD vs. HC, MCI vs. HC, AD vs. MCI) to determine the specific direction of intergroup differences.

**Table 2:** Protein expression analysis among three groups.

Proteins	Groups			*F*-value	*p*-value
	AD	MCI	HC		
4E-BP1	7.59 ± 0.87^*^	7.85 ± 0.60^#^	8.83 ± 0.57	8.96	0.001
ADA	5.28 ± 0.49^*^	5.32 ± 0.17^#^	6.01 ± 0.78	5.74	0.008
ARTN	−1.23 ± 0.37	−1.23 ± 0.39	−1.24 ± 0.26	0.00	0.998
AXIN1	3.71 ± 1.35	3.64 ± 0.95	4.38 ± 0.77	1.51	0.240
Beta-NGF	1.38 ± 0.13	1.33 ± 0.10	1.43 ± 0.13	1.66	0.208
CASP-8	1.09 ± 0.56^*^	1.04 ± 0.36^#^	4.75 ± 1.34	61.04	0.000
CCL11	7.58 ± 0.49	7.42 ± 0.39	7.51 ± 0.44	0.30	0.742
CCL19	10.28 ± 1.00	10.14 ± 0.79	10.14 ± 0.91	0.08	0.921
CCL20	8.34 ± 0.84	8.10 ± 0.97	8.50 ± 0.86	0.50	0.610
CCL23	11.39 ± 0.40	11.23 ± 0.50	11.21 ± 0.36	0.54	0.591
CCL25	5.91 ± 0.47	6.07 ± 0.63	5.85 ± 0.48	0.43	0.652
CCL28	1.69 ± 0.49	1.50 ± 0.58	1.44 ± 0.51	0.64	0.533
CCL3	5.59 ± 0.47^*^	5.68 ± 0.54	6.24 ± 0.70	3.72	0.037
CCL4	6.18 ± 0.64^*^	6.26 ± 0.58	7.00 ± 0.84	4.25	0.025
CD244	4.96 ± 0.36	4.84 ± 0.45	4.90 ± 0.25	0.25	0.780
CD40	10.68 ± 0.50	10.52 ± 0.40	10.31 ± 0.42	1.73	0.197
CD5	5.12 ± 0.36	5.02 ± 0.40	5.34 ± 0.25	2.25	0.125
CD6	4.42 ± 0.38^*^	4.53 ± 0.43^#^	5.11 ± 0.62	5.74	0.008
CD8A	9.66 ± 0.62	9.49 ± 0.94	9.10 ± 0.57	1.55	0.231
CDCP1	3.92 ± 0.45*	3.93 ± 0.65^#^	3.19 ± 0.46	6.46	0.005
CSF-1	9.43 ± 0.40	9.29 ± 0.24	9.20 ± 0.19	1.57	0.227
CST5	6.98 ± 0.66	7.02 ± 0.79	6.89 ± 0.53	0.10	0.906
CX3CL1	3.68 ± 0.30*	3.62 ± 0.59	3.20 ± 0.30	3.79	0.035
CXCL1	8.35 ± 0.80	7.95 ± 0.81	7.87 ± 1.67	0.47	0.628
CXCL10	8.18 ± 0.53	8.68 ± 1.10	8.36 ± 0.76	0.93	0.408
CXCL11	7.38 ± 0.78	7.62 ± 1.11	7.91 ± 0.61	0.97	0.394
CXCL5	10.22 ± 1.47	9.91 ± 1.71	10.26 ± 1.71	0.13	0.876
CXCL6	7.30 ± 0.96	7.26 ± 0.73	7.16 ± 1.38	0.05	0.954
CXCL9	7.21 ± 0.64	7.48 ± 1.49	6.88 ± 0.85	0.82	0.451
DNER	8.83 ± 0.23	8.83 ± 0.28	8.73 ± 0.24	0.52	0.598
EN-RAGE	1.15 ± 0.84*	1.30 ± 0.53^#^	4.71 ± 0.45	102.09	0.000
FGF-19	8.94 ± 1.15	8.51 ± 1.28	8.57 ± 0.88	0.43	0.654
FGF-21	4.71 ± 1.55	4.90 ± 1.86	5.87 ± 1.47	1.46	0.250
FGF-23	0.93 ± 0.37	1.07 ± 0.50	0.84 ± 0.54	0.61	0.551
FGF-5	1.31 ± 0.31	1.18 ± 0.29	1.05 ± 0.27	2.05	0.149
Flt3L	9.34 ± 0.45*	9.09 ± 0.35	8.71 ± 0.62	4.35	0.023
GDNF	1.55 ± 0.43	1.54 ± 0.34	1.21 ± 0.63	1.61	0.219
HGF	7.97 ± 0.54	8.03 ± 0.31	8.11 ± 0.29	0.29	0.754
IFN-gamma	7.56 ± 1.48	8.17 ± 2.39	7.65 ± 0.83	0.38	0.686
IL-1 alpha	−1.39 ± 0.61	−1.65 ± 1.57	−1.67 ± 0.37	0.74	0.487
IL10	2.44 ± 0.55	2.39 ± 2.30	2.05 ± 0.53	1.15	0.330
IL-10RA	−0.03 ± 1.14	−0.15 ± 0.05	0.04 ± 1.24	0.08	0.925
IL-10RB	5.72 ± 0.24	5.50 ± 0.60	5.60 ± 0.30	0.70	0.507
IL-12B	6.27 ± 0.54	5.93 ± 0.62	5.83 ± 0.53	1.68	0.205
IL13	−0.93 ± 1.32	−1.75 ± 0.34	−1.71 ± 0.41	3.22	0.056
IL-15RA	0.07 ± 0.30	0.02 ± 0.31	−0.04 ± 0.33	0.28	0.758
IL-17A	−0.21 ± 0.73	−0.36 ± 0.68	−0.40 ± 0.60	0.22	0.808
IL-17C	1.89 ± 0.72	2.23 ± 1.17	1.92 ± 0.51	0.50	0.611
IL18	8.83 ± 0.42*	8.78 ± 0.45^#^	12.54 ± 0.82	133.32	0.000
IL-18R1	6.45 ± 0.32	6.56 ± 0.39	6.42 ± 0.34	0.44	0.648
IL2	−1.17 ± 0.26	−1.26 ± 0.37	−1.26 ± 0.20	0.36	0.699
IL-20	−0.37 ± 0.34	−0.62 ± 0.21	−0.450.15	2.66	0.088
IL-20RA	−0.58 ± 0.41	−0.43 ± 0.52	−0.79 ± 0.32	1.78	0.188
IL-22 RA1	−0.26 ± 0.44	−0.26 ± 0.38	−0.53 ± 0.62	0.98	0.387
IL-24	0.19 ± 0.47	0.08 ± 0.48	0.68 ± 1.31	1.43	0.256
IL-2RB	−0.66 ± 1.16	−0.86 ± 0.46	−0.95 ± 0.35	0.38	0.686
IL33	−0.41 ± 0.36	−0.48 ± 0.26	−0.27 ± 0.53	0.74	0.486
IL4	−1.04 ± 0.62	−1.15 ± 0.66	−1.04 ± 1.68	0.03	0.969
IL5	−1.54 ± 0.58	−1.10 ± 0.85	−1.05 ± 0.75	1.31	0.286
IL6	2.34 ± 0.73	2.33 ± 0.81	2.60 ± 1.16	0.27	0.765
IL7	0.37 ± 0.54	0.06 ± 0.49	0.68 ± 0.65	2.99	0.067
IL8	5.17a ± 0.60	5.27a ± 0.85	8.12b ± 1.76	20.20	0.000
LAP TGF-beta-1	5.50 ± 0.46	5.19 ± 0.39	5.53 ± 0.47	1.82	0.182
LIF	−1.38 ± 0.19	−1.36 ± 0.36	−1.18 ± 0.42	1.04	0.368
LIF-R	2.81 ± 0.25	2.78 ± 0.31	2.60 ± 0.22	1.89	0.170
MCP-1	11.17 ± 0.59	10.99 ± 0.38	10.72 ± 0.37	2.36	0.113
MCP-2	8.28 ± 0.82	8.24 ± 0.94	8.44 ± 0.74	0.16	0.852
MCP-3	0.97 ± 0.82	1.04 ± 0.62	1.17 ± 0.39	0.24	0.785
MCP-4	14.35 ± 0.72	14.27 ± 0.37	14.20 ± 0.85	0.13	0.880
MMP-1	12.85 ± 1.27*	13.00 ± 1.23^#^	14.28 ± 0.94	4.64	0.018
MMP-10	7.79 ± 0.31	7.66 ± 0.65	7.75 ± 0.71	0.13	0.882
NRTN	−0.78 ± 0.90	−1.19 ± 0.35	−0.97 ± 0.20	1.24	0.304
NT-3	3.10 ± 0.66	3.03 ± 0.51	2.75 ± 0.48	1.09	0.350
OPG	10.15 ± 0.29	10.05 ± 0.34	9.93 ± 0.40	1.03	0.369
OSM	4.19 ± 0.99*	4.02 ± 0.76^#^	5.22 ± 0.52	6.87	0.004
PD-L1	4.77 ± 0.37	4.72 ± 0.40	4.52 ± 0.41	1.15	0.331
SCF	9.08 ± 0.25^^^	8.79 ± 0.24	8.67 ± 0.28	6.71	0.004
SIRT2	2.86 ± 1.30*	2.81 ± 0.81	4.03 ± 1.02	4.23	0.025
SLAMF1	1.30 ± 0.37	1.10 ± 0.67	1.02 ± 0.42	0.82	0.450
ST1A1	1.76 ± 0.93*	1.98 ± 0.80^#^	3.32 ± 0.62b	11.29	0.000
STAMBP	4.27 ± 0.88	4.23 ± 0.61	4.93 ± 0.71	2.84	0.076
TGF-alpha	2.98 ± 0.73*	2.68 ± 0.48^#^	3.79 ± 0.51	9.67	0.001
TNF	2.91 ± 0.60	2.74 ± 0.54	2.96 ± 092	0.28	0.756
TNFB	3.83 ± 0.16	3.78 ± 0.32	3.78 ± 0.30	0.12	0.888
TNFRSF9	5.41 ± 0.48	5.35 ± 0.59	5.49 ± 0.37	0.21	0.812
TNFSF14	3.82 ± 0.83*	3.74 ± 0.51^#^	5.11 ± 0.81	10.95	0.000
TRAIL	7.96 ± 0.22	8.08 ± 0.40	7.79 ± 0.47	1.41	0.262
TRANCE	3.27 ± 0.60	3.38 ± 0.93	3.68 ± 0.68	0.78	0.467
TSLP	−1.25 ± 0.61	−1.65 ± 0.66	−1.52 ± 0.50	1.15	0.331
TWEAK	9.12 ± 0.16	9.06 ± 0.26	8.93 ± 0.22	2.01	0.057
uPA	10.54 ± 0.34*	10.39 ± 0.23^#^	9.90 ± 0.24	14.80	0.000
VEGFA	11.22 ± 0.54	11.14 ± 0.43	11.42 ± 0.65	0.70	0.505

AD, Alzheimer’s disease; MCI, Mild cognitive impairment; HC, Healthy Control. Data are presented as mean ± standard deviation. Three groups’ proteins were compared using one-way ANOVA, post hoc Tukey’s test. ^*^p < 0.05 AD vs. HC; ^#^*p* < 0.05 MCI vs. HC, ^^^*p* < 0.05 AD vs. MCI.

### 3.3 AD vs. HC

**Figure 3:**
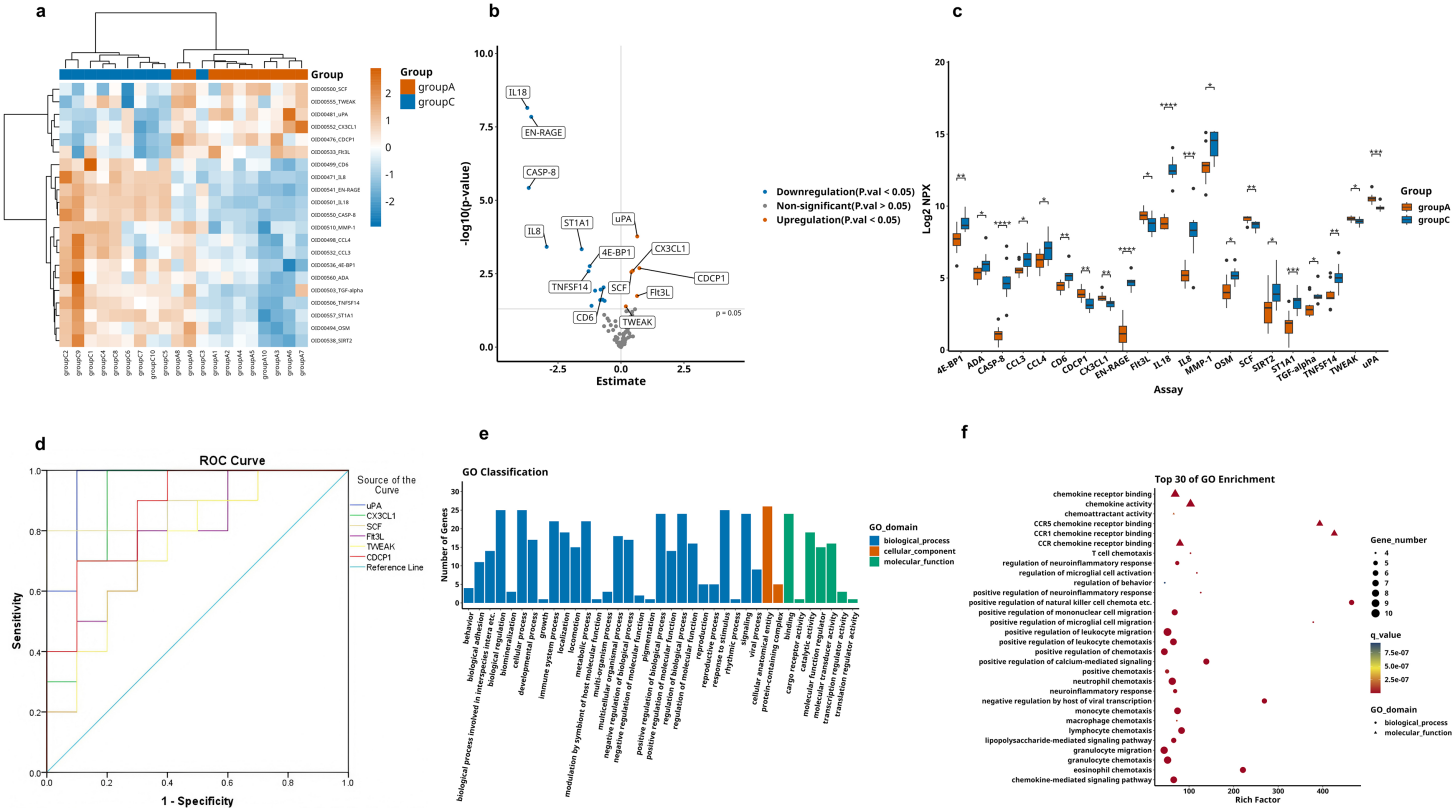
Comparison of DEPs between AD group and HC group, where A represents AD and C represents HC. **(a)** Expression patterns of DEPs between group A and group C, orange represents group A and blue means group A, darker colors indicate higher values, while lighter colors denote lower values. **(b)** Volcano plot showing 21 significantly DEPs between group A and C, red indicates upregulation, blue indicates downregulation, and gray represents no statistical significance. **(c)** Box plots of proteins with significant differences between group A and group C. **(d)** ROC curves for upregulated proteins: uPA, CX3CL1, CDCP1, SCF, Flt3L, and TWEAK. **(e,f)** GO analysis of the group A and group C, displaying functional distribution bar charts and enrichment bubble plots (^*^p < 0.05, ^**^p < 0.01, ^***^p < 0.001, ****p < 0.0001). These comparisons are exploratory and should be interpreted with caution due to potential Type I error.

**Figure 4:**
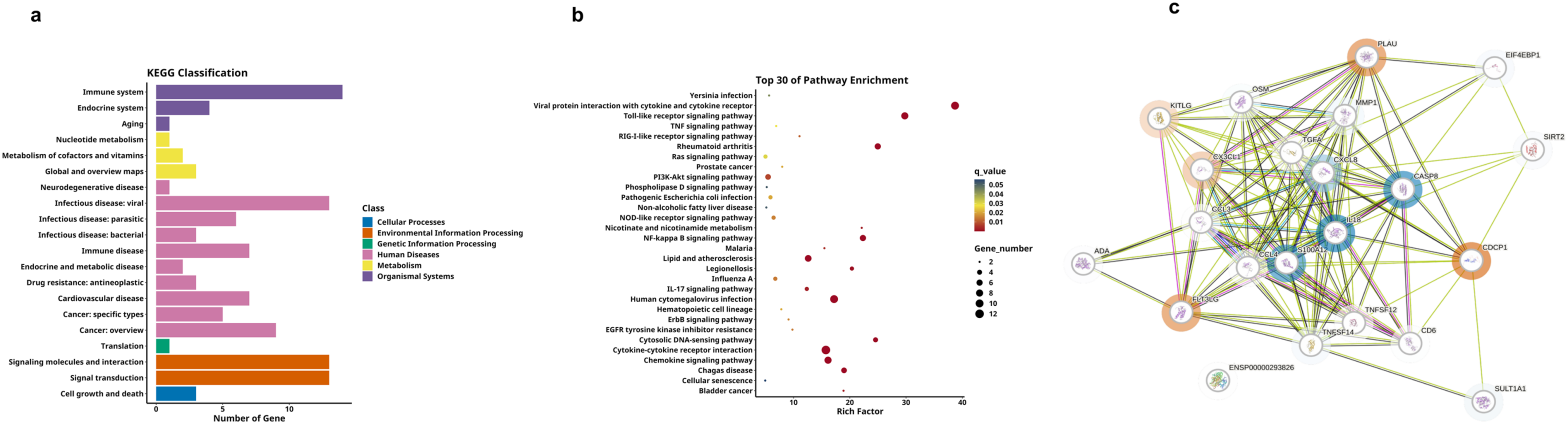
KEGG analysis and DEPs interaction network of group A and C. **(a,b)** Respectively display the functional distribution bar chart and enrichment bubble chart of KEGG pathways associated with differentially expressed protein-coding genes. **(c)** PPI network of DEPs between group A and group C. The blue circles represent downregulated DEPs, red circles denote upregulated DEPs, and proteins without circles indicate non-DEPs, the darker the color, the greater the inter-group difference in NPX values, and the size of the circle indicates the degree of connectivity of the protein.

Comparative analysis revealed significantly differential proteins between AD and HC groups, as detailed in Table 3. The heatmap specifically showed the hierarchical clustering of the DEPs (Figure 3a). Additionally, the volcano plot (Figure 3b) revealed that, compared to the HC group, a total of 21 inflammation-related proteins showed differential expression, including 6 up-regulated proteins (uPA, CX3CL1, CDCP1, SCF, Flt3L, TWEAK) and 15 down-regulated proteins. To more intuitively display the expression levels of these differential proteins, we used box plots for specific display (Figure 3c). Subsequently, for these DEPs, we performed ROC curve analysis (Figure 3d), which yielded the following results: the AUC of CDCP1[*p* = 0.005, AUC = 0.870, 95%CI (0.714–1.000), sensitivity of 90%, specificity of 70%]; the AUC of CX3CL1[*p* = 0.002, AUC = 0.900,95%CI (0.751–1.000), sensitivity of 80%, specificity of 90%], AUC for Flt3L[*p* = 0.018, AUC = 0.770, 95%CI (0.560–0.980), sensitivity of 80%, specificity of 70%], AUC for SCF[*p* = 0.003, AUC = 0.890, 95%CI (0.734–1.000), sensitivity of 80%, specificity of 90%], AUC for TWEAK[*p* = 0.041, AUC = 0.750, 95%CI (0.533–0.967), sensitivity of 90%, specificity of 50%], and AUC for uPA [*p* = 0.001, AUC = 0.960, 95%CI (0.875–1.000), sensitivity of 90%, specificity of 90%]. Their optimal cutoff values were 3.391 pg./mL, 3.411 pg./mL, 9.038 pg./mL, 8.984 pg./mL, 8.998 pg./mL, 10.083 pg./mL, respectively (Table 4). We performed GO and KEGG enrichment analyses to explore the potential functions of DEPs in the AD group. In GO analysis, chemokine activity, regulation of neuroinflammatory response, regulation of microglia activation, regulation of positive chemotaxis, and cytokine-mediated signaling pathways were enriched (Figures 3e,f). Meanwhile, KEGG pathway analysis revealed enrichment of cytokine-cytokine receptor interactions, human cytomegalovirus infection, and NK-Kappa B signaling pathway (Figures 4a,b). Finally, to investigate the interactions among DEPs, we analyzed by protein–protein interaction (PPI) network analysis and found that CDCP1 might be a core protein (Figure 4c).

**Table 3:** Analysis of differentially expressed proteins among three groups.

Proteins	*p*-value	*p*-adjust	*p*-posthoc
AD vs. HC			
IL18	0.001	0.000	0.001
EN-RAGE	0.000	0.000	0.001
CASP-8	0.000	0.000	0.001
uPA	0.000	0.004	0.001
IL8	0.000	0.007	0.001
ST1A1	0.000	0.007	0.001
4E-BP1	0.002	0.023	0.001
CDCP1	0.002	0.023	0.011
CX3CL1	0.002	0.023	0.037
TNFSF14	0.003	0.023	0.001
SCF	0.003	0.023	0.004
CD6	0.009	0.071	0.010
MMP-1	0.011	0.072	0.026
TGF-alpha	0.011	0.072	0.001
OSM	0.012	0.073	0.005
Flt3L	0.018	0.104	0.018
ADA	0.024	0.128	0.014
CCL4	0.025	0.128	0.034
CCL3	0.027	0.128	0.042
SIRT2	0.039	0.179	0.040
TWEAK	0.041	0.044	NA
MCI vs. HC			
EN-RAGE	0.000	0.000	0.001
IL18	0.000	0.000	0.001
CASP-8	0.000	0.000	0.001
TGF-alpha	0.000	0.002	0.012
uPA	0.000	0.004	0.001
TNFSF14	0.000	0.006	0.002
IL8	0.000	0.006	0.001
ST1A1	0.001	0.007	0.002
OSM	0.001	0.009	0.018
4E-BP1	0.001	0.013	0.009
SIRT2	0.009	0.072	NA
CDCP1	0.010	0.074	0.012
MMP-1	0.018	0.127	0.039
ADA	0.021	0.140	0.022
CD6	0.028	0.156	0.034
IL7	0.028	0.156	NA
STAMBP	0.029	0.156	NA
CCL4	0.035	0.180	NA
AD vs. MCI			
SCF	0.018	0.042	0.043

AD, Alzheimer’s disease; MCI, Mild cognitive impairment; HC, Healthy Control. The *p*-values were derived from t-tests, while p-adjust represents the Benjamini-Hochberg corrected *p*-values for proteins that showed significant differences in t-tests. The post-hoc *p*-values were obtained from pairwise comparisons of proteins that demonstrated significant differences in one-way ANOVA. NA indicated no statistically significant difference.

**Table 4:** Comparative ROC analysis of inflammatory biomarkers in three patient groups.

Variable	AUC	95%CI		*p*-value	Cut-off values	Sensitivity	Specificity
		Minimum value	Maximum value				
AD vs. HC							
CDCP1	0.870	0.714	1.000	0.005	3.391 pg./mL	0.900	0.700
CX3CL1	0.900	0.751	1.000	0.002	3.411 pg./mL	0.800	0.900
Flt3L	0.770	0.560	0.980	0.018	9.038 pg./mL	0.800	0.700
SCF	0.890	0.734	1.000	0.003	8.984 pg./mL	0.800	0.900
TWEAK	0.750	0.533	0.967	0.041	8.998 pg./mL	0.900	0.500
uPA	0.960	0.875	1.000	0.001	10.083 pg./mL	0.900	0.900
MCI vs. HC							
CDCP1	0.830	0.633	1.000	0.013	3.803 pg./mL	0.800	0.900
uPA	0.920	0.767	1.000	0.001	10.133 pg./mL	0.900	0.900
AD vs. MCI							
SCF	0.840	0.658	1.000	0.010	8.984 pg./mL	0.800	0.800

AUC, Area Under the ROC Curve; CI, Confidence Interval; AD, Alzheimer’s disease; MCI, Mild cognitive impairment; HC, Healthy Control. With statistical significance observed at *p* < 0.05.

### 3.4 MCI vs. HC

**Figure 5:**
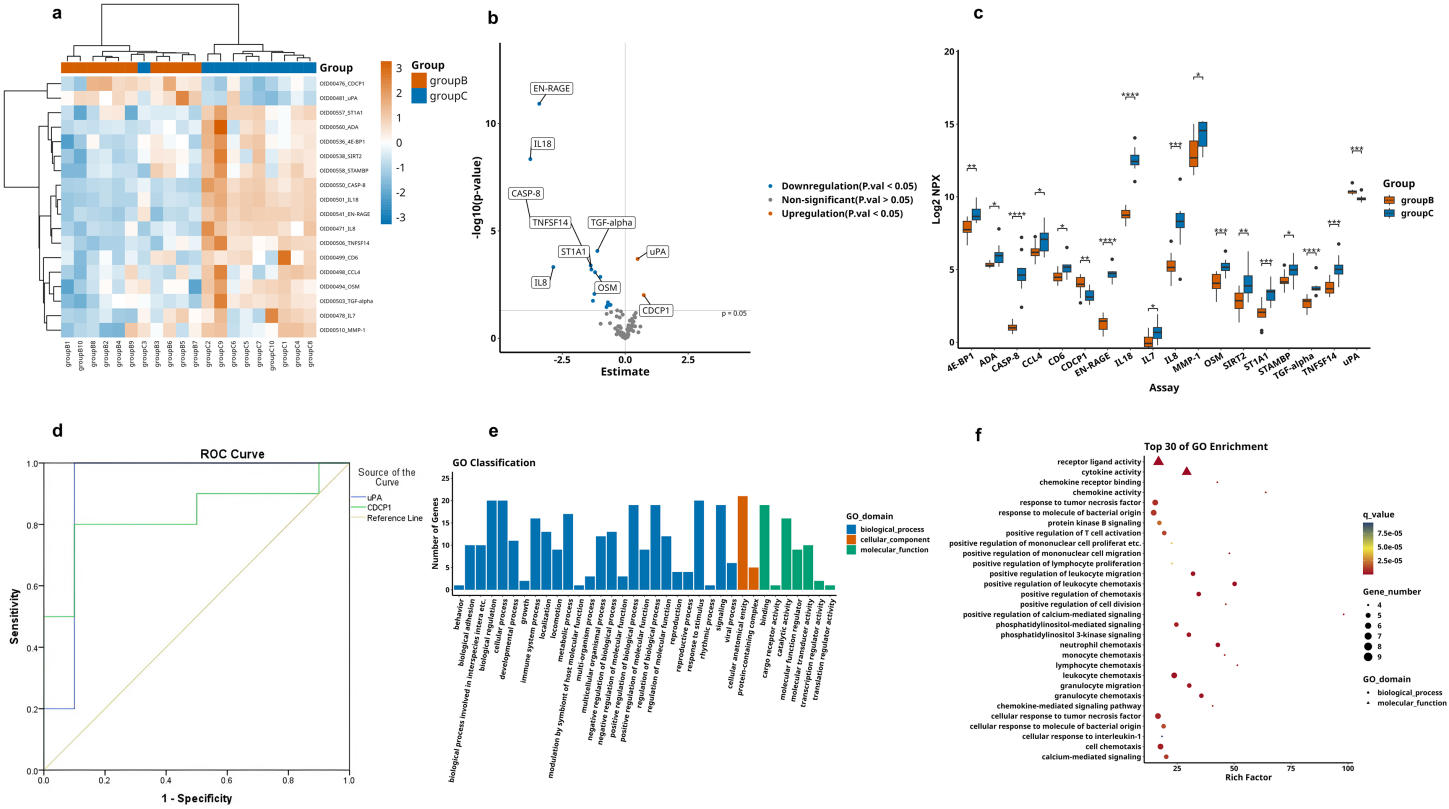
Comparison of DEPs between the MCI group and HC group, where B represents MCI and C represents HC. **(a)** Heatmap showing expression levels of DEPs between group B and group C. group B is represented by orange, and group C by blue, the darker color, the higher value. **(b)** Volcano plot illustrating the 18 significantly DEPs shared between group B and C, red denotes upregulation, blue denotes downregulation, and gray indicates no statistical significance. **(c)** Box plots comparing the expression levels of significantly DEPs between group B and group C. **(d)** ROC curves for upregulated proteins uPA and CDCP1. **(e,f)** GO analysis of the group B and group C, displaying functional distribution bar charts and enrichment bubble plots (^*^p < 0.05, ^**^p < 0.01, ^***^p < 0.001). These comparisons are exploratory and should be interpreted with caution due to potential Type I error.

**Figure 6:**
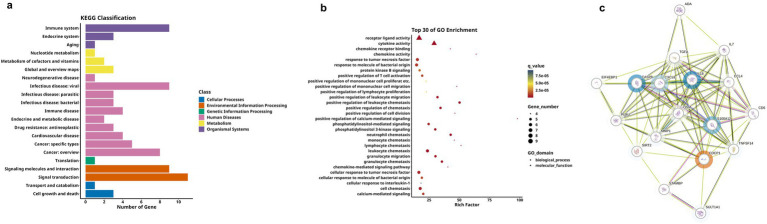
KEGG analysis and DEPs interaction network for group B and C. **(a,b)** Respectively display the bar chart and enrichment bubble chart showing the functional distribution of KEGG pathways associated with the DEPs-coding genes. **(c)** PPI network of DEPs between group B and group C, where blue circles represent downregulated DEPs, red circles represent upregulated ones, and proteins without circles indicate non-DEPs, the deeper the color, the greater the inter-group difference in NPX values, and the size of the circle indicates the degree of connectivity of the protein.

We next analyzed differences between the MCI and the HC group, as shown in Table 3. Firstly, the hierarchical clustering heatmap showed the DEPs (Figure 5a). Through volcano plot (Figure 5b), we found that there were 16 differentially expressed inflammation-related proteins in MCI group compared to HC group, of which 2 were up-regulated (uPA, CDCP1) and 12 were down-regulated (Figure 5c). The results of the ROC curve analysis (Figure 5d) showed that the AUC of CDCP1 [*p* = 0.013, AUC = 0.830, 95%CI (0.633–1.000), sensitivity of 80%, specificity of 90%], and the AUC of uPA,[*p* = 0.001, AUC = 0.920, 95%CI (0.767–1.000), sensitivity of 90%, specificity of 90%]. Their optimal cutoff values were 3.803 pg./mL, 10.133 pg./mL, respectively (Table 4). In addition to this, we performed GO and KEGG enrichment analysis to investigate the potential function of DEPs in the MCI group. In GO analysis, receptor ligand activity, cytokine activity, cellular chemotaxis was enriched (Figures 5e,f). KEGG pathway showed phosphatidylinositol-mediated signaling, cytokine-cytokine receptor interactions, human cytomegalovirus infection, and chemokine signaling pathway enrichment (Figures 6a,b). We attempted to study the interactions of DEPs and showed by PPI network that CDCP1 might be the core protein (Figure 6c).

### 3.5 AD vs. MCI

**Figure 7:**
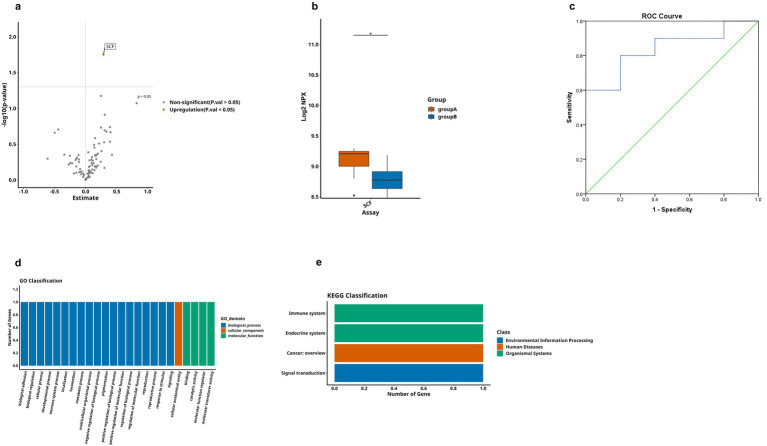
Comparison of DEPs between AD and MCI groups, where A represents AD and B represents MCI. **(a)** The volcano plot demonstrates that only one DEP exists between group A and B. **(b)** Boxplot showing significantly DEPs between group A and group B. **(c)** ROC curve for upregulated protein SCF. **(d,e)** Bar charts illustrating the functional distributions of GO and KEGG pathways associated with the differentially expressed protein-coding genes in group A and group B, respectively. (* < 0.05). These comparisons are exploratory and should be interpreted with caution due to potential Type I error.

Finally, we conducted a comparative analysis of the expression levels of DEPs between AD group and MCI group, as shown in Table 3. Through observation of the volcano plot (Figure 7a), compared with the MCI group, only one inflammation-related protein (SCF) was found to show an upregulation trend in AD group, and the box plot showed that this difference was statistically significant (Figure 7b). For further exploration, ROC curve analysis was performed (Figure 7c), which showed that the AUC of SCF [*p* = 0.010, AUC = 0.840, 95%CI (0.658–1.000), sensitivity of 80%, specificity of 80%]. It had an optimal cutoff value of 8.984 pg./mL (Table 4). Meanwhile, we also performed GO analysis and KEGG pathway analysis on the DEPs (Figures 7d,e).

## 4 Discussion

AD is the most common irreversible neurodegenerative disease, with its incidence increasing year by year, imposing a heavy health, economic and social burden worldwide. For decades, numerous clinical trials have been carried out for AD (23), focusing on different AD targets, but unfortunately, almost all of them ended in failure (24–26). Up to now, only three effective targeted drugs, namely Lecanemab, Donannan, and Aducanumab, have been approved by the Food and Drug Administration (FDA) of the United States for the treatment of AD (27, 28). This state of affairs amply demonstrates that in-depth exploration of the pathological process of AD and the discovery of new potential therapeutic targets remains a daunting and far-reaching task. Recent studies have shown that neuroinflammation plays a crucial role in the occurrence and development of AD (29, 30). However, most of the current studies on AD inflammation-related proteomics have mainly focused on CSF or brain tissue, with relatively less attention paid to peripheral plasma. Although some studies have identified several proteins associated with AD risk through plasma proteomic studies (31), such as GFAP (32), Neurofilament light polypeptide (NEFL), and neurosecretory protein Nerve Growth Factor (VGF) (33), systematic analyses of neuroinflammation-related peripheral plasma between AD patients and healthy individuals are still scarce (19). It has been shown that in adults with normal cognitive function, increased plasma inflammatory markers are strongly associated with reduced brain volume, declining cognitive potential, and an increased risk of dementia decades later (34). In this study, we quantitatively compared 92 plasma inflammatory proteins among AD patients, MCI patients, and healthy controls, identifying significant DEPs. First, compared with the healthy group, the levels of uPA and CDCP1 proteins both showed an increasing trend in AD patients and MCI patients. Secondly, in addition to the two proteins mentioned above, the expression levels of another four proteins (CX3CL1, SCF, Flt3L, and TWEAK) were also significantly elevated in the AD patients. Lastly, compared with the MCI patient group, except for the upregulation of SCF expression, no downregulated proteins were found in the AD patient group.

CDCP1 (CUB domain-containing protein 1), also referred to as CD318, is a type I single transmembrane glycoprotein containing three CUB structural domains. As an immunoinflammatory marker, CDCP1 enhances the activation and infiltration of T cells, which in turn leads to an increase in the production of pro-inflammatory cytokines (35). Numerous studies have shown that CDCP1 is closely associated with AD, and its expression level shows a significant positive correlation with the risk of AD onset. Mitogen-activated protein kinases (MAPKs), as a type of serine/threonine protein kinase, are mainly composed of three signaling pathways: extracellular signal-regulated kinase 1/2 (ERK1/2), Jun N-terminal kinases (JNKs), and p38-MAPK. By phosphorylating serine or threonine residues in proteins, they play key roles in regulating processes such as cell survival, differentiation, proliferation, and apoptosis. In the brain, the MAPK/ERK pathway can transfer signals from the cell membrane to the nucleus, which is deeply involved in the regulation of neuronal proliferation and apoptosis, and is closely linked to the function of learning and memory, which is intricately involved in the pathophysiological mechanisms of AD (36). Specifically, during the development of AD, this signaling pathway participates in Aβ-dependent neurotoxicity. Fibrillar Aβ induces ERK1/2 activation, and sustained ERK1/2 activation leads to abnormal phosphorylation of tau proteins, which in turn triggers progressive neurodegeneration and cell death (37). In addition, in the CNS, activation of p38 MAPK has also been found to be associated with phenomena such as excessive phosphorylation of Tau protein, release of pro-inflammatory factors, memory dysfunction, and neuronal apoptosis (38) In a phase II study of neuroprotective agents for MCI, the expression of inflammatory proteins such as CDCP1 and TWEAK in the CSF of patients was significantly reduced after treatment (39). Recent studies have also found that CDCP1 is a protein strongly associated with inflammatory dietary patterns and adverse neurocognitive outcomes (40), plasma levels of CDCP1 were elevated in response to long-term inflammatory dietary pattern interventions, which were positively correlated with the Aβ42/40 ratios and were related to Aβ accumulation and neurodegeneration. Meanwhile, CDCP1 can effectively distinguish some dementia cases from cognitively normal elderly people (31). Prior studies have established that inflammatory proteins (41, 42) contributed to AD pathogenesis via MAPK signaling pathways. We therefore hypothesized that CDCP1 may similarly interface with AD through this mechanistic axis. In our study, the protein expression levels of CDCP1 were significantly elevated in patients in the cognitively impaired groups (AD and MCI patients), suggesting that dynamic fluctuations in CDCP1 levels may mirror disease progression in AD. Future studies could develop a risk stratification model utilizing CDCP1 cutoff values to identify high-risk cohorts warranting early therapeutic intervention. Furthermore, given the observed correlation between CDCP1 and Aβ pathology, CDCP1 holds promise as a potential biomarker for monitoring treatment efficacy, particularly in the context of Aβ-targeted therapies such as aducanumab. This finding provided new ideas for predicting the progression of AD and was expected to provide a theoretical basis for future targeted pathway therapies.

CX3CL1 protein, also known as Fractalkine, is a chemotactic cytokine that is widely expressed in hippocampal and cortical neurons. It affects the pathological process of AD by binding to the receptor CX3CR1, which is located on the surface of microglia, and thus has an impact on the pathological process of AD (43). Under physiological conditions, the interaction between CX3CL1 and CX3CR1 exerts anti-inflammatory efficacy by inducing microglia to maintain a resting state while promoting the synthesis of anti-inflammatory cytokines (44). In the early stage of AD, the expression level of CX3CL1 in plasma is significantly higher than that in the severe AD stage. This may be due to the aggregation of Aβ protein stimulating microglial cells and neurons, prompting the latter to secrete CX3CL1, which achieves neuroprotection by recruiting microglia and enhancing their phagocytosis capacity to remove Aβ (45, 46). Subsequently, in the development process of AD, intra-neuronal Aβ accumulates gradually, which triggers the down-regulation of the CX3CL1/CX3CR1 signaling pathway when the accumulation reaches a certain threshold. This change in turn promotes the occurrence and development of neuroinflammation, impairs the uptake and degradation ability of Aβ, contributes to a further increase in Aβ deposition, and accelerates the phosphorylation process of tau proteins (47). During the development of AD, the expression level of CX3CL1 was negatively correlated with the severity of AD. Specifically, in the plasma of patients with mild AD, the expression level of CX3CL1 shows an increasing trend, and then as the disease deteriorates to the inflammatory stage, its expression level decreases significantly (48). Given the complex pathogenesis of AD, CX3CL1 may exert neuroprotective effects or be neurotoxic at different stages of disease evolution. At the same time, the association between CX3CL1 and microglial activation underscores its dual role as both a diagnostic marker for neuroinflammatory regulation and a potential therapeutic target. In our study, the expression level of CX3CL1 in patients with early AD increased. A plasma CX3CL1 protein concentration level >3.411 pg./mL demonstrated a sensitivity of 80% (AUC = 0.900) in discriminating AD. In the future, we plan to combine its measurement with core biomarkers (5), which is expected to improve diagnostic accuracy while reducing costs. Furthermore, the elevation of CX3CL1 exclusively in the AD population supports its utility as an independent staging biomarker, which means that by rapid testing of CX3CL1 protein levels may enable earlier patient stratification. Therefore, implementing early intervention measures before this change occurs may effectively delay the disease progression of AD.

uPA (Urokinase-type plasminogen activator) protein is one of the components of the fibrinogen activation system, playing an important role in physiological and pathological processes such as cell differentiation, migration, and tissue remodeling. During the progression of AD, uPA also plays a pivotal role. In the central nervous system, Aβ oligomers significantly up-regulate the expression of uPA and its specific receptor (uPAR) in human cerebrovascular smooth muscle cells through the activation of ERK1/2 signaling pathway (49). Notably, uPA promotes axonal and synaptic recovery after various forms of brain injury by activating the plasminogen-plasmin cascade (50). This mechanism effectively protects cortical neurons from synaptic damage induced by soluble amyloid-beta. However, as the disease progresses, an excessive increase in Aβ levels leads to a decrease in uPA synaptic expression (51), which in turn loses its protective effect. In our study, the uPA protein showed a trend of up-regulated expression in early AD compared to the healthy population, which corresponds to the protective mechanism one.

As an important hematopoietic growth factor, Flt3L (FMS-like tyrosine kinase 3 ligand) protein plays a key role in hematopoiesis by specifically binding to the Flt3L receptor, which in turn activates a series of downstream signaling pathways (52). Meanwhile, as a crucial differentiation factor, Flt3L protein can effectively promote the neural activity of microglia. In a previous study, researchers used a multiplex Enzyme-Linked Immunosorbent Assay (ELISA) technique for multiple immune-related proteins, with gray matter tissue of the anterior cingulate cortex and cerebrospinal fluid as samples, to conduct a comparative analysis of various neurodegenerative diseases, including AD. The results showed that Flt3L was specific for AD and had the highest association with AD among all the proteins tested (53). However, to the best of our knowledge, as of now, no study has reported the association between plasma Flt3L levels and AD. In the present study, we demonstrated that the expression level of Flt3L was significantly higher in AD patients than in healthy controls, suggesting that it may play a certain role in the pathogenesis of AD. In addition, we determined 9.038 pg./mL as the critical value of Flt3L for predicting AD versus healthy individuals. This finding suggests that plasma levels of Flt3L may serve as a potential biomarker for AD, showing significantly differences from healthy subjects. Future studies should allow further exploration of the mechanism of Flt3L protein’s role in pathophysiological processes such as inflammatory response and angiogenesis, as well as its potential application value in the treatment of AD.

TWEAK (Tumor necrosis factor-like weak inducer of apoptosis), as a member of the tumor necrosis factor (ligand) superfamily, it has been proven in clinical research to have pro-apoptotic activity against specific tumor cells and is very likely to be involved in the pathogenesis of chronic inflammatory diseases. TWEAK proteins are not only associated with cognitive function but is also closely related to the risk of AD (54). Until now, no study has been able to clearly elucidate the intrinsic association mechanism underlying their association. However, in a study on an animal model of neuropsychiatric lupus, it was observed that TWEAK plays a facilitating role in disrupting the blood–brain barrier and triggering neuronal damage caused by hippocampal glial cell proliferation (55). We found that there were differences in the expression of TWEAK between participants with AD and HC group, which suggests that they may share the same mechanism of action, and this may provide a brand-new reference direction for the subsequent development of AD animal models. It should be emphasized that in our study, although the TWEAK protein did not reach overall significance in the ANOVA (*p* = 0.057), the t-test between the AD and HC groups showed a significant increase (p-adjust = 0.044), suggesting that this protein may be associated with late-stage disease progression. Further validation with larger sample sizes is warranted.

Similar to Flt3L protein, SCF (Stem Cell Factor) protein also belongs to the category of hematopoietic factors. It has the ability to increase in the immune cell population of the brain, and such macrophages which can take up and degrade aggregated Aβ (56). This mechanism of action plays an important role in the process of AD. Relevant studies have found that SCF can inhibit L-glutamate-induced endoplasmic reticulum stress-related apoptosis in primary hippocampal neurons of AD model mice by means of the JAK2/STAT3 axis (57). In AD patients, SCF showed a protective effect and its level was inversely related to the severity of AD. However, this is somewhat contradictory to the increased SCF expression we observed in the early stage of AD. Previous studies have shown (58) that microglia play a “double-edged sword” role in the development of AD. On the one hand, microglia can clear Aβ deposits, which is neuroprotective. On the other hand, their chronic activation induces neuroinflammation, exacerbating neuronal damage (59). Based on AD’s complex pathogenesis, we hypothesize that SCF protein may undergo a compensatory process during disease progression. In early AD, SCF expression will increase to enhance brain immune capacity and improve anti-inflammatory levels. Therefore, the number of macrophages, microglia, etc., will continuously increase and become activated, thereby engulfing Aβ fibrils. However, these activated cells will also continuously release inflammatory factors, accelerating neuronal apoptosis. Although SCF itself has anti-apoptotic properties, an imbalance favoring microglial dominance shifts the net effect toward apoptosis. Accompanied by lasting inflammatory response, SCF expression progressively declines in mid-to-late AD stages. This loss of compensatory capacity leads to uncontrolled Aβ deposition and disease progression. It has been reported (60) that stem cell factor supplementation improves symptoms of AD in a mouse model and that the intervention has a favorable safety profile. This research achievement provides a potential therapeutic direction for AD treatment. Nevertheless, at the current stage, relevant clinical trials have not been carried out yet, and such treatment methods have not been further verified and applied. Furthermore, so far, no relevant research has been reported on the compensatory process we proposed this time. In future research, we will further promote this research work to obtain more experimental data and results, so as to further verify the hypothesis we put forward.

Of interest, during the course of this study, we found a correlation between the virus and the occurrence of AD, an important finding that is in line with numerous previous studies demonstrating that viral infections can lead to an increased risk of AD and other neurodegenerative diseases (61, 62). In the present study, we found that cytomegalovirus-dominated inflammatory pathways are involved in the early development of AD. Cytomegalovirus is an extremely common virus, and its infection is very prevalent among the population, with all people having the possibility to be infected with the virus. However, only a small number of people has a chronically active state of the virus in the gut. Recently, it has been shown (63) that cytomegalovirus can activate microglia through the complex physiological mechanism of the gut-brain axis, keeping them in a continuously activated state. These continuously activated microglia will trigger a series of pathophysiological changes, specifically manifested as increasing the contents of Aβ amyloid protein and tau protein, and further promoting the degeneration and death processes of neurons. These findings make us need to view AD from a brand-new perspective.

Although the aim of this study was to compare differential plasma inflammatory proteins in patients with AD, MCI and healthy individuals, to determine their cut-off values and to predict their potential as a potential biomarker for AD, it cannot be ignored that there are some limitations in our experiment. First of all, the relatively limited sample size may have reduced statistical power, increasing the risk of Type II errors. Besides, the single-center cross-sectional design precluded causal inferences regarding plasma inflammatory markers and AD and may limit population representativeness, while limitations in data availability prevented comprehensive adjustment for potential confounders (e.g., comorbidities, medications) that could independently influence inflammatory protein levels. In the future, multi-center studies with diverse demographics and longitudinal follow-up are warranted to validate our observations. Additionally, our proteomic analysis did not apply multiple comparison correction [MCC, (e.g., FDR or Bonferroni)], which increased the risk of false-positive findings. Although we reported nominally significant results (*p* < 0.05) to maximize sensitivity for hypothesis generation, these results should be interpreted with caution until replicated in larger cohorts and MCC. In the end, our analytical approach to the MCI subgroup requires refinement. In this study, we prioritized the inclusion of memory-predominant cognitive impairment based on clinically diagnosed criteria. However, the exclusion of non-AD pathologies (e. g., dementia with Lewy bodies) relied solely on clinical evaluations rather than biomarker confirmation. This methodology may introduce elevated false-positive rates due to potential misclassification of etiologically heterogeneous MCI cases. Notwithstanding these limitations, our study provided evidence supporting the association between systemic inflammation and AD, and identified several proteins as promising inflammatory protein candidates for further investigation, which have not been sufficiently studied in previous research. To validate the clinical utility of these inflammatory proteins, we plan to conduct a community-based cohort study in Xuzhou, China. This study will enroll participants using standardized clinical assessments (NIA-AA criteria), reduce the influence of confounding factors, with an expanded sample size (at least >1,000) and age-stratified analysis. We will establish cutoff values based on ROC curve analysis (with prespecified sensitivity and specificity > 75% for each) and evaluate their clinical utility indices. Longitudinal follow-up data (>2 years) will be collected to develop predictive models and assess the prognostic value of these cutoff values for conversion to AD.

## 5 Conclusion

Our research found that inflammatory proteins show differential expression in the plasma of patients with AD and MCI. The levels of CDCP1, CX3CL1, uPA, Flt3L and TWEAK protein in plasma could have the value of serving as potential biomarkers for AD patients. Moreover, the findings suggest there may be a potential etiological link between viral infections and the pathogenesis of AD. Although our study has certain limitations, such as a relatively small sample size and a cross-sectional design, the findings remain of great clinical significance for the early detection of AD and the implementation of targeted interventions or treatments. At the same time, they also provide new ideas and research directions for further exploring the inflammation-related pathogenesis of AD and screening potential therapeutic targets.

## Data Availability

The datasets presented in this study can be found in online repositories. The names of the repository/repositories and accession number(s) can be found at: https://figshare.com/, 10.6084/m9.figshare.28829720.
